# The Detached Mindfulness approach to anxiety disorders in an Italian mental health service

**DOI:** 10.1192/j.eurpsy.2024.789

**Published:** 2024-08-27

**Authors:** F. Raffone, E. Pessina, A. Martini, P. Giunnelli, A. Massa, E. Carbone, M. Russo, V. Martiadis

**Affiliations:** ^1^Department of Mental Health, Asl Napoli 1 Centro, Napoli; ^2^Department of Mental Health, Asl Cuneo 2, Bra, Italy

## Abstract

**Introduction:**

Anxiety disorders are one of the most common mental illnesses, and a consistent increase was observed after the COVID-19 pandemic. Mindfulness refers to a process that leads to a mental state characterized by nonjudgmental awareness of the present experience. Mindfulness can be considered both a skill and a practice. The stronger is the ability to adopt a mindful state, the less suffering one will experience. While Mindfulness-based Psychotherapies have shown efficacy in their treatment, they have not yet been thoroughly studied in Italian public mental health services. In Detached Mindfulness, negative thoughts are acknowledged and avoided by turning them into actions using a standardized, time-limited, metacognitive intervention.

**Objectives:**

The purpose of this study is to examine the efficacy and cost-effectiveness of Detachment Mindfulness for twelve patients with Generalized Anxiety Disorder (GAD) not being treated pharmacologically.

**Methods:**

We enrolled 12 patients diagnosed with GAD according to DSM-V in an 8-session program of Detached Mindfulness Psychotherapy (once a week). The Generalized Anxiety Disorder - 7 Scale (GAD-7) and the Kellner Symptom Questionnaire (SQ) were used to assess anxiety symptoms at baseline (T0), after 4 sessions (T1), and at the end of treatment (T2). The Client Satisfaction Questionnaire (CSQ-8) was used to assess treatment satisfaction.

**Results:**

The GAD-7 score showed consistent reductions in generalized anxiety symptoms after Detached Mindfulness treatment (mean decrease of -42% at the end of the program). As measured by SQ, patients also reported improvement in physical well-being, relaxation, and somatic symptoms significantly respect to baseline. As for treatment satisfaction, ten out of twelve patients rated their treatment as satisfactory. As reported by patients, mindfulness can become a powerful and effective means to relate to one’s own internal experiences such as anxiety or fear, learning to recognize them, staying with them and avoiding their consequences.

**Conclusions:**

These results showed that detached mindfulness was an effective and cost-effective intervention for GAD, given the short amount of time it requires and the ease with which it can be implemented. For its extensive use in the public mental health system to be further supported, studies on larger populations are needed.

**Disclosure of Interest:**

None Declared

